# Dynamics of Social Interaction: Kinematic Analysis of a Joint Action

**DOI:** 10.3389/fpsyg.2016.02016

**Published:** 2016-12-27

**Authors:** Quentin Moreau, Lucie Galvan, Tatjana A. Nazir, Yves Paulignan

**Affiliations:** ^1^“Sapienza” Università di Roma, Dipartimento di PsicologiaRoma, Italy; ^2^Laboratoire sur le Langage, le Cerveau et la Cognition, Institut des Sciences Cognitives, Centre de National de la Recherche Scientifique – Université Claude-Bernard Lyon 1Lyon, France; ^3^Istituto di Ricovero e Cura a Carattere Scientifico, Fondazione Santa Lucia, Laboratorio di Neuroscienze SocialiRoma, Italy

**Keywords:** joint action, kinematics and dynamics, social interactions, reach-to-grasp, motor system

## Abstract

Non-verbal social interaction between humans requires accurate understanding of the others’ actions. The cognitivist approach suggests that successful interaction depends on the creation of a shared representation of the task, where the pairing of perceptive and motor systems of partners allows inclusion of the other’s goal into the overarching representation. Activity of the Mirror Neurons System (MNS) is thought to be a crucial mechanism linking two individuals during a joint action through action observation. The construction of a shared representation of an interaction (i.e., joint action) depends upon sensorimotor cognitive processes that modulate the ability to adapt in time and space. We attempted to detect individuals’ behavioral/kinematic change resulting in a global amelioration of performance for both subjects when a common representation of the action is built using a repetitive joint action. We asked pairs of subjects to carry out a simple task where one puts a base in the middle of a table and the other places a parallelepiped fitting into the base, the crucial manipulation being that participants switched roles during the experiment. We aimed to show that a full comprehension of a joint action is not an automatic process. We found that, before switching the interactional role, the participant initially placing the base orientated it in a way that led to an uncomfortable action for participants placing the parallelepiped. However, after switching roles, the action’s kinematics by the participant who places the base changed in order to facilitate the action of the other. More precisely, our data shows significant modulation of the base angle in order to ease the completion of the joint action, highlighting the fact that a shared knowledge of the complete action facilitates the generation of a common representation. This evidence suggests the ability to establish an efficient shared representation of a joint action benefits from physically taking our partner’s perspective because simply observing the actions of others may not be enough.

## Introduction

Humans are constantly communicating with their fellows. Of all the great apes, it was only humans who developed a complex verbal language allowing us to communicate our wishes, our intentions and our feelings. Leibniz (1765) described language as the *mirror of understanding*, a powerful instrument used by an individual to express their own internal processes and to describe external objects for others and consequently socially interact with them. However, social communication is not limited to just verbal communication. Through their behavior, humans change their environment, satisfy internal needs, and achieve personal goals (Lemon, 2008). From our early days as infants, a large number of our actions are performed in social contexts, allowing us to develop social skills and the ability to coordinate with interacting agents. One way to study these behaviors is to focus on joint actions (Sebanz et al., 2006). Classical orchestras, collective sports, and ballets are just a few examples of how people can coordinate their movements to achieve a common goal. But such actions are not only accomplished by musicians or those competing in team sports. Simple joint actions are ubiquitous in our everyday life, such as lifting a heavy table with another person or shaking hands with a colleague. Social interactions require dynamic and efficient encoding of others’ gesture and a spatiotemporal synchronization of the individuals involved (Sebanz et al., 2006). On the question of the mechanisms of interaction, differing views have been proposed over the years. For example, Coordination Dynamics have explored the social influence of one person on another, highlighting a spontaneous and immediate coordination of their actions while engaging in interpersonal sensorimotor interactions (see Coey et al., 2012 for a review). This theory places social cognition in an embodied-embedded constraint, where social behavior is defined as self-organized. The brain is dynamically in interaction with the environment and other natural sytems (Coey et al., 2012). On the other hand, the most traditional approach of social cognition is set in a cognitivist framework. Evidence indicating that action production and action perception rely on common mechanisms led to the Theory of Event Coding (Hommel et al., 2001). If perception and action rely on common codes, it makes the integration of one’s own and co-actor’s action for joint actions relatively straightforward (bottom-up processes). On a more top-down perceptive, co-representation of conspecifics during joint action is thought to be a key feature to understanding others’ goals and actions (Sebanz et al., 2006).

One cerebral network suggested to play a role in matching observed and executed actions is the Mirror Neurons System (MNS). Since mirror neurons (MN) were discovered more than 20 years ago, we have been able to apply a neuroscientific approach and a new understanding of social interaction. Initially discovered in non-human primates (di Pellegrino et al., 1992) and in humans (Buccino et al., 2001; Rizzolatti and Craighero, 2004; Mukamel et al., 2010), MN are visuo-motor neurons located in the premotor cortex and the inferior parietal lobule, automatically firing during the execution of an action and the observation of the same action performed by another person. MNS has been proposed as the fundamental neural mechanism at the basis of the understanding of others’ actions and successful non-verbal social interactions (Gallese et al., 2004). The discovery of MN has given rise to a large number of interpretations of their potential roles in human cognition: understanding of action (Rizzolatti et al., 2001), imitation (Gallese, 2003), empathy (Gallese, 2001, 2003), mind-reading (Gallese and Goldman, 1998), and emergence of language (Rizzolatti and Arbib, 1998).

In recent years, great progress has been made by investigating neural processes during interpersonal motor interactions (Newman-Norlund et al., 2007; Newman-Norlund et al., 2008; Kokal and Keysers, 2010; Konvalinka et al., 2014; Ménoret et al., 2014; Sacheli et al., 2015b). These studies brought to light the recruitment of fronto-parietal networks during interactive contexts where MN are thought to play a role in the internal simulation, action prediction and understanding. However, interpersonal coordination requires both perception and understanding of our partner’s movements while controlling our own movements. To explain neural processes in bidirectional interactions, Hari and Kujala (2009) proposed the “interactive-loop” model. This model is that a coupling of perceptive and motor systems of the individuals is necessarily involved during joint actions in order to form common internal representations. By their actions and intentions, a person influences and changes not only their environment but also the movements that the interactional partner needs to perform in order to interact smoothly. These modifications modulate the perception of the environment the other person has had up to this point. That person reciprocates by influencing the environment in return by changing external representations on their partner’s brain. The progressive construction of these “action-perception” loops is constantly changing and seems to be essential for encoding social actions and building up a common representation of the action for all the protagonists involved (Hari and Kujala, 2009). The enrolment of fronto-parietal networks might therefore allow the construction of an “interactive loop” to build a common representation of an action in both protagonists, allowing successful interactions.

In line with this model, it is our opinion that building experimental paradigms allowing bidirectional and either synchronic or diachronic adaption (Tognoli and Kelso, 2015) involving at least two interacting subjects (Tognoli et al., 2007; Noy et al., 2011; Konvalinka et al., 2014; Sacheli et al., 2015a; Moreau and Candidi, 2016), it should be possible to highlight a common adaptation between participants at a behavioral level. In our study, we focused on behavior changes during a bidirectional diachronic joint-action, involving one participant putting a base in the middle of a table and another participant placing a parallelepiped into the base’s slot. Crucially, the action of placing the parallelepiped is more or less facilitated depending on how the base is oriented on the table (for an equivalent solo action, see Allami et al., 2008). Our paradigm therefore allows space for adaptation and gives us objective measures to define the success of the interaction and the installation of the common representation. The purpose of the study, set on a cognitivist approach, is to highlight that full comprehension of a joint action is not automatic and that the installation of the common representation is progressive.

We attempted to reveal a behavioral change (placing the base in a more optimal position) when both individuals had *physically experienced* the other partner’s motor task difficulty. Results indicate that the adaptation to the new task was not automatic and required a common experience from both participants. We wish to point out a limit of the maximalist interpretations of the MNS, according to which this system serves the understanding of others action and provide empirical data supporting the minimalist approach of the representational content of MN (Pacherie and Dokic, 2006). Although we agree the MNS is part of basic constituents of action understanding, this system appeared not sufficient to fully understand actions and apprehend movement difficulties encountered by the other.

## Materials and Methods

### Subjects

Twenty healthy right-handed subjects (6 males, 14 females; mean age: 22.9, range : 18–50 years) participated in the Main experiment and 10 healthy right-handed subjects participated in the Control Experiment (5 males, 5 females; mean age : 21.2, range : 16–28 years). None of them had any history of neurological disorder. All participants gave written consent before the experiment. Participants were familiarized with the methods prior to the experiment. and an explanation of the purpose of the study was given at the end of the experiment. The study was approved by the Ethical Committee CPP Sud-Est II.

### Procedure

#### Main Experiment

**FIGURE 1:**
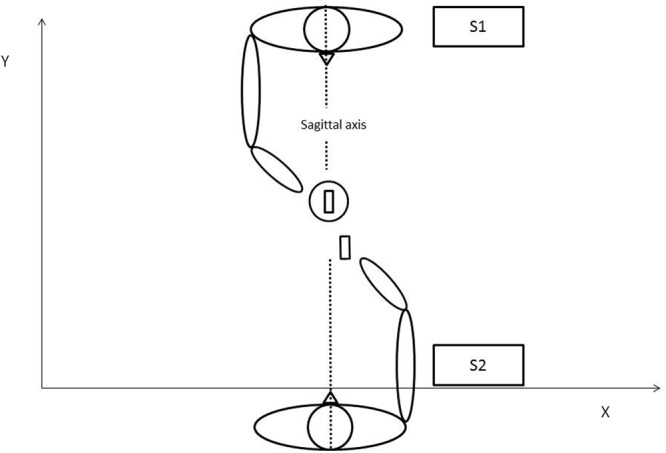
**Schematic representation of the joint action task in Condition 1**.

The experiment involved a pair of participants: subject 1 (S1) and subject 2 (S2). In the following, participants will be referred as S1 or S2 depending on their original role in the experiment (Condition 1). Subjects were seated face to face on opposite sides of a 50 cm × 70 cm table. Both subjects were instructed to use only their thumbs and index fingers to grip and displace the experimental objects, which were placed on predefined spots: the cylindrical base located to the right of S1 and the parallelepiped to the right of S2. The slot of the base and the parallelepiped were both oriented parallel to the sagittal axis (**Figure 1**). The subjects were asked to carry out a simple task: S1 was to move the base to the center of the table and S2 was to place the parallelepiped in the slot on the base. Note that the action performed by S2 is more or less facilitated depending on how S1 place the base on the table: difficult when the slot in the base remains parallel to the sagittal axis; easy when it is slightly tilted to the left. The experiment was divided into three conditions with instructions given prior to each condition. In Condition 1, the joint action was repeated 20 times with no other instruction other than completing the task after a vocal “Go” signal from the experimenter, which was the same for every trial. In Condition 2, subject’s roles were interchanged so S1 was in charge of moving the parallelepiped and S2 the base for 3 trials. Because this second condition was designed to give the opportunity for participants to experience the other’s action, data from Condition 2 was not analyzed. In Condition 3, subjects reverted to their initial roles (so they were carrying out the same task as in Condition 1) for another 20 trials. In this final condition, participants were further instructed to perform the task as fast as possible. During the whole experiment, participants were not allowed to communicate their feelings either verbally or by explicit non-verbal utterance. To ensure the accuracy and well-being of the experiment, an experimenter continuously stayed next to the table in order to both control inter-subjects communication and to replace objects at their correct initial location when required. At no time were participants encouraged to change their motor behavior either explicitly or implicitly; they believed the goal was a simple kinematic study until the end of the experiment.

#### Control Experiment

In the Control Experiment, the task was explained as in the Main Experiment but subjects only completed Condition 3 (where the task was to perform the joint action as fast as possible). This control served to ascertain that verbal instruction to perform the task as fast as possible was not sufficient to provoke potential behavioral changes. A critical point of the Control experiment was that participants were only assigned to one role; they did not have the opportunity to experience the other participant’s task.

### Movements Recording

The movements of the right arm and hand of both subjects were recorded by means of an Optotrak Certus camera (manufactured by Northern Digital Inc, in Waterloo, Ontario, Canada). The camera was fixed 2 m away from the table. Five markers were placed on each subject: marker 1 was on the distal extremity of the thumb, marker 2 on the distal extremity of the index, marker 3 on the metacarpophalangeal joint of the index, marker 4 on the radial styloid and marker 5 was 3 cm over the radial styloid. The spatial position of active markers was sampled at 250 Hz with a spatial precision of 0.1 mm.

Raw data was pre-processed using a second order Butterworth dual-pass filter (cut-off frequency, 10 Hz). Kinematic parameters were assessed for each individual movement using Optodisp software (Optodisp copyright INSERM-CNRS-UCBL, Thévenet et al., 2001). The movement duration was analyzed and the velocity peak of the two sub-phases of the movement recorded (*Reach-to-Grasp* and *Displace*). Sub-movements onset and offset were determined by a sequence of at least eleven increasing or decreasing points of the wrist marker velocity profiles. Velocity peak was determined as the maximal value in wrist marker velocity profiles. The workspace was defined by the X, Y, Z axes defined by the table surface. The angle between the fingers markers 1 and 2 of S2 and the sagittal axis was also analyzed. This angle corresponded to the opposition axis (Paulignan et al., 1997) an index of movement difficulty (Frak et al., 2001). Note that the opposition axis is directly linked to the behavior of the subject that places the slot (S1) because the opposition axis changes with the orientation of slot.

### Statistical Analyses

Kinematic parameters were determined for each individual trial and averaged for each participant and condition. Statistical analysis data was analyzed by using Statsoft Statistica 8. General Linear Model (GLM) and the Greenhouse–Geisser correction for non-sphericity was applied when appropriate (Keselman and Rogan, 1980). *Post hoc* comparisons were performed using the Newman–Keuls correction for multiple comparisons (significance threshold was fixed at *p* < 0.05).

Execution time and Velocity peaks were both analyzed each by separated 2×2×2 (Condition × Movement × Subject) ANOVAs and final angles by a one-way ANOVA.

## Results

### Kinematics Results

#### Execution Times

**FIGURE 2:**
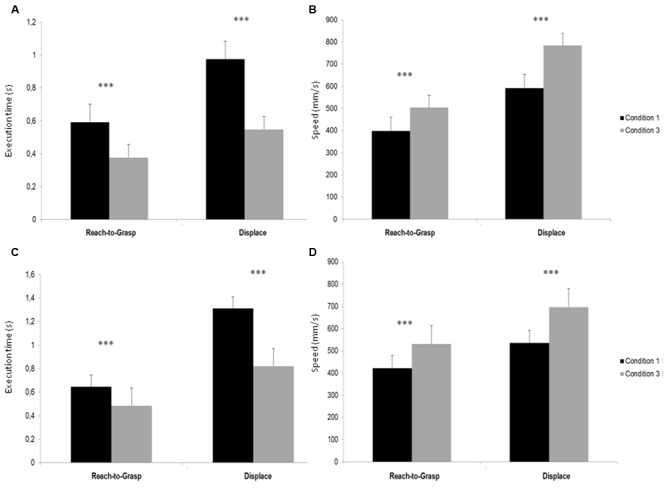
**(A)** Execution times (s) of *Reach-to-Grasp* and *Displace* S1’s Sub-movement in Conditions 1 and 3. **(B)** Execution times (s) of *Reach-to-Grasp* and *Displace* S2’s Sub-movement in Conditions 1 and 3. **(C)** Peak velocity (mm/s) of *Reach-to-Grasp* and *Displace* S1’s Sub-movement in Conditions 1 and 3. **(D)** Peak velocity (mm/s) of *Reach-to-Grasp* and *Displace* S2’s Sub-movement in Conditions 1 and 3. ^∗∗∗^*p* < 0.001.

The 2 × 2 × 2 (Condition × Movement × Subject) ANOVA showed a significant main effect of Condition [*F*(1,8) = 70,824, *p* < 0,001], indicating a smaller execution time during Condition 3 compared to Condition 1. There was a significant main effect of movement [*F*(1,8) = 111,06, *p* < 0,001], showing that *Reach-to-Grasp* movement was realized faster than *Displace* and a significant main effect of subject [*F*(1,8) = 59,335, *p* < 0,001] that showed that S1’s action were executed faster than S2’s. *Post hoc* test indicated that S1 execution times were smaller in Condition 3 compared to Condition 1 for both Reach-to-Grasp (*p* < 0,001) and Displace (*p* < 0,001) (see **Figure 2A**) and that S2 execution times were smaller in Condition 3 compared to Condition 1 for both Reach-to-Grasp (*p* < 0,001) and Displace (*p* < 0,001) (see **Figure 2B**).

#### Velocity Peaks

The 2 × 2 × 2 (Condition × Movement × Subject) ANOVA showed a significant main effect of Condition [*F*(1,8) = 49,339, *p* < 0,001], indicating a greater execution speed during Condition 3 compared to Condition 1. A significant main effect of movement [*F*(1,8) = 114,47, *p* < 0,001] showed that Reach-to-Grasp movement was realized with a smaller speed than Displace. Post hoc test indicated that S1 velocity peaks were greater in Condition 3 compared to Condition 1 for both Reach-to-Grasp (*p* = 0,006) and Displace (*p* < 0,001) (see **Figure 2C**) and S2 velocity peaks were greater in Condition 3 compared to Condition 1 for both Reach-to-Grasp (*p* = 0,002) and Displace (*p* < 0,001) (see **Figure 2D**).

### Final Angle

**FIGURE 3:**
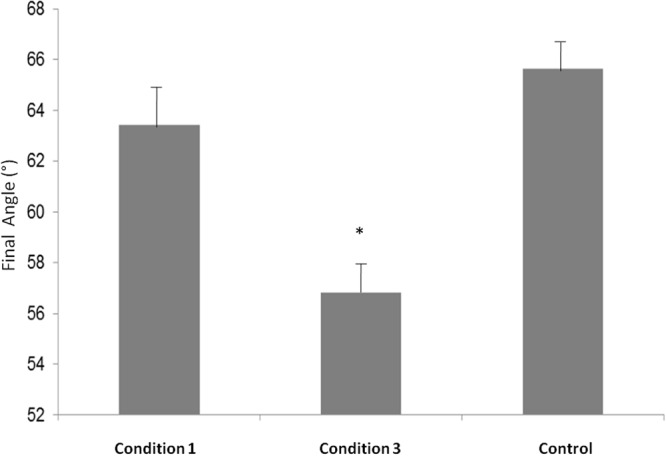
**Final Angle measures (°) in Condition 1, Condition 3 and Control.** ^∗^*p* < 0.05.

In order to compare the final angles of Condition 1, Condition 2, and Control, we performed a one way ANOVA that showed a main effect [*F*(2,6) = 7,7206, *p* = 0,02]. *Post hoc* test revealed that the final angle in Condition 3 was significantly smaller compared to Condition 1 (*p* = 0,03) and compared to Control (*p* = 0,02) (see **Figure 3**).

## Discussion

In this paper, we developed a diachronic joint action task that induced behavioral adjustments made by one of the participants in order to make the task of their partner easier and consequently improving the common realization of the task for both participants. The results revealed better performance (execution time and velocity) in both sub-movements of the task, *Reach-to-Grasp* and *Displace*, for both participants when common representation of the task was achieved.

Our measurements also highlight a significant change in the final angle of the cylindrical base between the Condition 1 (before the subjects switched roles) and Condition 3 (after the subjects switched their roles). According to Allami et al. (2008), this orientation’s change from the sagittal axis facilitates the partner *Displace* sub-movement: the articular tension in the arm and wrist joints are less extreme in Condition 3 than in Condition 1. This behavioral measurement indicates that the participant placing the base changed their previous behavior, after experiencing the partner’s contribution to the joint action.

The results from our statistical analysis reveal an increase in the performance of the subjects for both sub-movements during Condition 3. In Condition 3, both subjects were asked to perform the action as fast as possible. Due to this, one can assess that the instruction (to perform the task as fast as possible) is the main contributor to the increase in performance, rather than changes in the orientation. Because of this, we were also able to calculate the effect size (Cohen’s *d*) according to Fitts’s (1954) law, where the level of difficulty of a movement is proportional to the amplitude of kinematics values (Ferri et al., 2011). Thus we compared Cohen’s *d* values calculated for velocity peaks for each sub-movement in Condition 1 and Condition 3 for S2 (the participant placing the parallelepiped). Cohen’s *d* value for *Reach-to-Grasp* sub-movement between Condition 1 and Condition 3 is 0.76, corresponding to a medium effect, while Cohen’s d for *Displace* Sub-movement between Condition 1 and Condition 3 is 1.38, corresponding to a large effect (Cohen, 1992). The discrepancy between Cohen’s *d* values during the two movements’ phases highlights the facilitation of S2 *Displace* sub-movement when S1 (participants placing the base) changed the orientation of the base. On the one hand, imposed constrain on movement speed in Condition 3 improves the kinematics parameters in both sub-movements but on the other hand, we observe a greater orientation-specific effect only on the *Displace* sub-movement. This result is consistent with previous data from Allami et al. (2008).

Our aim, based on Hari and Kujala’s (2009) model, was to demonstrate the establishment of a common representation during a joint action. Through the first condition, the two subjects were performing the task freely. Even if Condition 1 was uncomfortable for the participant placing the parallelepiped (S2), the participant placing the base (S1) didn’t change the base’s orientation to facilitate S2 contribution. Nonetheless, after both subjects experienced each other’s role in the action (in Condition 2), a significant change in the base’s orientation has been measured, revealing a change in the behavior of S1 during Condition 3. This change also improved the task performance in *Displace* sub-movement of S2. Changes in opposition axis orientation are close to those obtained in previous studies describing easy movements (Frak et al., 2001; Allami et al., 2008).

As our Control experiment showed, the orientation change is not due to the requested high speed during Condition 3; indeed, no change in angle was measured when the roles of both subjects were not interchanged (Control condition). This change in the instructions was only to maintain participants focus and avoid boredom.

From the neural perceptive, parietal and premotor regions are thought to form the action observation network, also known as the MNS (Rizzolatti and Craighero, 2004). Although action mirroring has become a popular way to explain joint action effects (Frischen et al., 2009), if we adhere to the original description of this neural substrate, both action and observation should activate the same neurons. However, in our experiment, observation of S2’s action did not seem enough for S1 to fully understand their difficulty. In fact, S1 kept putting the base in a difficult position for S2 through all Condition 1. However, when S1 had experience of both parts of the joint-action, S1 adapted and changed their initial behavior to facilitate S2’s movement. We can agree here that MN were certainly activated to understand globally the action but not the subtleties such as extreme joint angles. The critical behavioral change only appeared in Condition 3, when both subjects shared a common knowledge of the two individual actions composing the joint action. In any case, our experiment stirs up a debate about MNS role in joint-actions. While we do not deny the possible role of MN for joint action, or the fact that they are involved in action comprehension, it seems that observation of the partner’s movements (and thus activity in the MNS) was not enough to fully understand the action of others (Kokal and Keysers, 2010) during our protocol. In fact, we highlighted a delayed installation of a common understanding between the two subjects participating in a joint action. Our interpretation of behavioral data is in line with Pacherie and Dokic (2006) minimalist theory.

Over the last few decades, scientific research has improved our understanding of how perception and action are linked. It still remains unclear whether the processing of relevant information during joint actions emerges from the physical and informational constraints (Rigoli et al., 2015) or whether it is supported by high-level representations (social-specific) mechanisms (Sebanz et al., 2006) or through lower perceptive mechanisms (Dolk et al., 2011). In other words, is the mental representation of a partner necessary, and if it is, to what extent does one interacting individual need to mentally represent the actions of others to interact with them?

One of the most studied joint tasks is derived from the Simon task (Simon, 1969) where participants have to respond to the color of stimuli with both their hands while ignoring their spatial location (e.g., red with left hand, blue with right hand). The so-called Simon effect (SE) describes the fact that participants are faster to answer when the stimulus and the response are congruent (e.g., use the left hand when the stimulus is presented of the left of the screen). The SE disappears when participants are asked to only answer to one stimulus (a go/no-go task) but the *joint* Simon effect arises when performed by a pair of participants (where each participant has a go/no-go task). The joint Simon task has been used to highlight co-representations during joint tasks, suggesting the existence of a specific neural mechanism facilitating social interactions with conspecifics (Tsai and Brass, 2007; Welsh, 2009). An alternative interpretation was proposed by Dolk et al. (2013), suggested that “social” effects from the Joint Simon task can be explained by the Theory of Event Coding (Hommel et al., 2001; Hommel, 2009) and further claimed that generic sensorimotor regions were the substrate of joint actions without the need of a co-representation. Another framework challenging the notion of co-representation during joint action is the Coordination Dynamics approach where social effects are due to motor coordination rather than mental stimulation of a complementary action (Doneva and Cole, 2014). The findings of our experiment seem to be contesting the latter theories (both Theory of Event Coding and Coordination Dynamics), rather they agree that the common representation of the action during social interaction is through mental representation. Our results appear to have a better fit in a top-down explanation: our behavioral change did not appear automatically through action observation nor motor coordination between subjects but only when knowledge of task-specific features was shared between the two participants. Although our conclusions are only based on kinematics, the change in the behaviors of participants seems to fit in the theory of co-representation. Here we argue that participants lacked knowledge of the others’ specific action, resulting in a misrepresentation during Condition 1. It is our belief that, only thanks to the shared experience of the task during Condition 2, was co-representation achieved, resulting in an adaptation of interacting subjects’ behaviors in Condition 3. In our task, the common understanding required both action perception and self-experienced execution of the subtasks in order to reach a common understanding through co-representation. Further experiments focusing on neural activity will be required in order to highlight the installation of the co-representation, such as reported modulations of the alpha rhythm during joint actions (Naeem et al., 2012; Novembre et al., 2016).

## Author Contributions

All authors provided substantial contributions to the conception or design of the work. QM and LG were in charge of the acquisition of data, QM, LG, and YP were in charge of the analysis, all authors were involved in the interpretation of data for the work. QM and YP drafted the work and TN revised it critically and all authors gave final approval of the version to be published.

## Conflict of Interest Statement

The authors declare that the research was conducted in the absence of any commercial or financial relationships that could be construed as a potential conflict of interest.
